# Characterization of iNOS^+^ Neutrophil-like ring cell in tumor-bearing mice

**DOI:** 10.1186/1479-5876-10-152

**Published:** 2012-07-30

**Authors:** Lauren P Virtuoso, Jamie L Harden, Paula Sotomayor, Wade J Sigurdson, Fuminobu Yoshimura, Nejat K Egilmez, Boris Minev, Mehmet O Kilinc

**Affiliations:** 1 Department of Microbiology and Immunology, School of Medicine and Biomedical Sciences, University at Buffalo, 3435 Main Street, Buffalo, NY, 14214, USA; 2 Department of Medicine, Roswell Park Cancer Institute, Buffalo, NY, USA; 3 Department of Microbiology School of Dentistry, Aichi-Gakuin University, Nagoya, Japan; 4 Genelux Corporation, San Diego, CA, USA; 5 Moores UCSD Cancer Center, University of California, San Diego, CA, USA; 6 Division of Neurosurgery, University of California, San Diego, CA, USA

**Keywords:** Myeloid-derived Suppressor Cells (MDSC), ring cell, inducible nitric oxide synthase (iNOS), Nitric oxide (NO), Neutrophil

## Abstract

**Background:**

Myeloid-derived Suppressor Cells (MDSC) have been identified as tumor-induced immature myeloid cells (IMC) with potent immune suppressive activity in cancer. Whereas strict phenotypic classification of MDSC has been challenging due to the highly heterogeneous nature of cell surface marker expression, use of functional markers such as Arginase and inducible nitric oxide synthase (iNOS) may represent a better categorization strategy. In this study we investigated whether iNOS could be utilized as a specific marker for the identification of a more informative homogenous MDSC subset.

**Methods:**

Single-cell suspensions from tumors and other organs were prepared essentially by enzymatic digestion. Flow cytometric analysis was performed on a four-color flow cytometer. Morphology, intracellular structure and localization of iNOS^+^ ring cells in the tumor were determined by cytospin analysis, immunofluorescence microscopy and immunohistochemistry, respectively. For functional analysis, iNOS^+^ ring subset were sorted and tested *in vitro* cell culture experiments. Pharmacologic inhibition of iNOS was performed both *in vivo* and *in vitro*.

**Results:**

The results showed that intracellular iNOS staining distinguished a granular iNOS^+^ SSC^hi^ CD11b^+^ Gr-1^dim^ F4/80^+^ subset with ring-shaped nuclei (ring cells) among the CD11b^**+**^ Gr-1^+^ cell populations found in tumors. The intensity of the ring cell infiltrate correlated with tumor size and these cells constituted the second major tumor-infiltrating leukocyte subset found in established tumors. Although phenotypic analysis demonstrated that ring cells shared characteristics with tumor-associated macrophages (TAM), morphological analysis revealed a neutrophil-like appearance as detected by cytospin and immunofluorescence microscopy analysis. The presence of distinct iNOS filled granule-like structures located next to the cell membrane suggested that iNOS was stored in pre-formed vesicles and available for rapid release upon activation. Tumor biopsies showed large areas with infiltrating ring cells primarily surrounding necrotic areas. Importantly, these cells significantly impaired CD8^+^ T-cell proliferation and induced apoptotic death. The intratumoral accumulation and suppressive activity of ring cells could be blocked through pharmacologic inhibition of iNOS, demonstrating the critical role of this enzyme in mediating both the differentiation and the activity of these cells.

**Conclusions:**

In this study, iNOS expression was linked to a homogeneous subset; ring cells with a particular phenotype and immune suppressive function, in a common and well-established murine tumor model; 4T-1. Since the absence of a Gr-1 homolog in humans has made the identification of MDSC much more challenging, use of iNOS as a functional marker of MDSC may also have clinical importance.

## Background

Myelocytic cells that are found in human and mouse tumors represent a heterogeous mixture of mature and immature myeloid cells (IMC) [reviewed in Refs. [1-3]]. IMCs arise from a differentiation process called myelopoiesis that takes place in the bone marrow. Under normal conditions IMCs differentiate into macrophages, dendritic cells (DC) and granulocytes. However, tumor-derived inflammatory factors prevent proper differentiation of IMC resulting in the appearance of a highly heterogeneous myeloid cell population with subsets arrested at different stages of development [2]. They are known to be activated by various factors secreted by tumor stroma and produce increased levels of Arginase (ARG) or iNOS, which have been associated with T cell suppression [3]. Because of this activity they are commonly referred as MDSC [4]. A distinct phenotypic marker that uniquely identifies MDSC among other myeloid cells has not been identified. Instead, CD11b and Gr-1 cell surface markers have been used for their identification in mice. However, this combination is not unique to MDSC and in addition to the phenotypic similarity, functional overlap has also been observed between the conventional myeloid cells and MDSC. Anti-Gr-1 antibody, which binds to the myeloid differentiation marker Gr-1, recognizes two epitopes, Ly6C and Ly6G. In subsequent studies two main subsets of MDSC, i.e. mononuclear (MO-) MDSC, which display a CD11b^+^ Ly6G^-^ Ly6C^hi^IL-4Rα^+^phenotype and polymorphonuclear (PMN-) MDSC, which have a CD11b^+^ Ly6G^+^ Ly6C^lo^IL-4Rα^+^phenotype, were identified [5,6]. In more recent studies the MDSC have been categorized into multiple subsets further complicating phenotypic classification. Greifenberg et al. divided CD11b^+^ Gr-1^+^ double positive (DP) myeloid cells into six different subsets according to their differential expression of Gr-1 and CD11b, identifying two different MO- and two PMN-MDSC populations all with suppressive function [7]. A year later, a study by Dolcetti et al. subdivided MDSC into 3 fractions of MDSC based on Gr-1 intensity; Gr-1^lo^, Gr-1^int^, Gr-1^hi^[8]. The same year Movahedi et al. showed at least seven tumor-infiltrating subsets and among those, 4 subsets could readily be distinguished based on the differential expression of Ly6C and MHCII. They recognized these subpopulations as TAM [9]. In another study, a novel marker, CD49d, was suggested as an alternative marker for Gr-1 to differentiate between the subpopulations of MDSC [10]. As evidence by all these recent studies, identification of an individual MDSC subset with a specific function has been difficult because of the lack of unique cell surface markers that can distinguish between different myeloid subtypes. Their classification was further complicated by the plasticity of MDSC [reviewed in Refs. [11,12]]. An example of the phenotypic switch among the myeloid cell population is that F4/80^+^ monocytes have been shown to be the precursors of functionally distinct subsets of TAM [9] and DC [13,14].

Although, recent studies have started to combine phenotypic characterization based on Ly6G/C staining with cytospin analysis and functional testing to further describe individual subpopulations of myeloid cells, a clear categorization strategy has not yet emerged. Therefore, the conflicting phenotypic descriptions of the populations necessitate further studies to sort out individual subsets based on functional markers associated with specific morphological and functional characteristics.

While staining with Gr-1 in mice, is becoming a generally accepted basis for evaluating MDSC, there is no corresponding counterpart to this in humans. The absence of a Gr-1 homolog has made the identification of human MDSC much more challenging. But it is generally agreed that they are suppressive with a CD33^+^, CD11b^+^, CD15^+^, HLA-DR^low/-^, CD14^+/−^ phenotype [15].

In this study we identified and characterized a homogeneous subset within the tumor- infiltrating CD11b^**+**^ Gr-1^+^cells using functional marker iNOS. This bone marrow (BM)-derived population expressed the monocyte/macrophage marker F4/80, accumulated rapidly in the growing tumor and the periphery, and constituted the second major tumor-infiltrating leukocyte subset. Further phenotypic characterization coupled with morphological analysis revealed that this subset consist of ring cells which phenotypically resembled TAM to some extent but morphologically were more akin to neutrophils. *In vitro* studies showed that the iNOS^+^ subset can inhibit T cell proliferation through the production of nitric oxide (NO) and induce their apoptosis. *In vivo* iNOS inhibition significantly repressed the accumulation of ring cells in the spleen and the tumor and concomitantly resulted in increased CD8^+^ T-cell numbers.

## Methods

### Mice, tumor induction and reagents

Six- to 8-wk-old BALB/c and C57BL/6 mice were purchased from Taconic Laboratories. Clone-4 mice that bear T cells transgenic for a HA- specific (IYSTVASSL) T-cell receptor and FVBneuN mice (FVB/N-TgN^MMTVneu^202Mul) were bred in the Laboratory Animal Facility of University at Buffalo [16]. The BALB/c syngeneic mammary carcinoma cell line 4T1 has been described [17]. CT26 colon carcinoma cell line was maintained in DMEM/F-12 (Invitrogen Life Technologies) supplemented with 10% heat-inactivated FBS (Equitech-bio), 2 mM L-glutamine, 100 U/ml penicillin, 100 μg/ml streptomycin (Mediatech). Same medium with an additional 2-mercaptoethanol was used for B16 cell line. Briefly, mice were injected s.c. with 0.5 × 10^6^-1 ×10^6^ viable tumor cells in 0.1 ml sterile PBS behind the neck just above the scapula. Tumors were allowed to reach a size of 350–450 mm^3^. For inhibition of iNOS activity, N6-(1-iminoethyl)-L-lysine, dihydrochloride (L-NIL) or 1,3-PB-ITU dihydrobromide (1,3-PB-ITU) was injected i.p. (0.2 mg/100 μl PBS) daily. At least five mice per group were analyzed. For the determination of the absolute number of specific cell populations, the percentage of each population was multiplied by the number of cells recovered from the respective tissue [18]. All animals were housed and treated according to NIH guidelines under the auspices of the UB IACUC.

### Preparation of single-cell suspensions, enrichment and fluorescence-activated cell sorting

Single-cell suspensions from tumors and other organs were prepared essentially by enzymatic digestion as previously described [18]. Bone marrow cells were obtained from the femurs and tibias [19]. To purify iNOS^+^ cells, single-cell suspensions were magnetically labeled with Anti-Ly-6G microBeads. Then, the cell suspension was loaded onto auto MACS in order to deplete Gr-1^hi^ Ly-6G^**+**^ cells (MiltenyiBiotec). Unlabeled cells ran through; this cell fraction was thus depleted of Gr-1^hi^ Ly-6G^**+**^ and pre-enriched for Gr-1^lo^ Ly-6G^**-**^ myeloid cells. Pre-enriched fraction was further enriched for F4/80^+^ subset using F4/80-PE along with anti-PE microBeads (Positive selection).In the final step, F4/80^+^ subset were further sorted using a BD FACSAria II (BD Biosciences) using SSC profile as distinguishing criteria among iNOS^+^ and iNOS^-^ subpopulations. The purity of the total SSC^hi^ F4/80^+^ iNOS^+^ population was typically higher than 95%.

### Flow cytometry

Flow cytometric analysis of single-cell suspensions prepared from tumors and other peripheral organs was performed on a four-color FACSCalibur flow cytometer (BD Pharmingen) using established protocols as previously described [18]. Fluorochrome-conjugated anti-mouse monoclonal Antibodies (mAbs) to iNOS (6/iNOS/NOS type II), Gr-1 (RB6-8 C5), CD11b (M1/70), Ly6C (AL-21), Ly6G (1A8), CD45 (30-F11),CD138 (281–2), CD193(CCR3/83103), CD54(ICAM-1/3E2), CD119 (IFNGR1/2E2), CD124 (mIL4R-M1), Flk-1 (VEGFR2/Avas12α1), CD14 (rmC5-3), Siglec-F (E50-2440) and all isotype controls were purchased from BD Pharmingen. Anti-CD184 (CXCR4/2B11), CD49d (R1-2), CD115 (CSFR1/AFS98), CD282 (TLR2/12-9021), F4/80 (BM8) were obtained from eBioscience and Anti-CD182 (TG11/CXCR-2) was purchased from Bio Legend. All other mAbs and intracellular iNOS staining were as described previously [20-22]. 7-AAD viability staining solution was purchased from BD Pharmingen.

### Cytospin analysis, immunofluorescence microscopy and Immunohistochemistry

Sorted cells were centrifuged in 200 ul PBS onto a microscope slide using a Cytospin 3 cytocentrifuge (Shandon Instruments, PA) and stained with Protocol Hema 3 kit (Fisher Diagnostics). Preparation of Alcian blue cover slips was done as previously described [23]. Briefly, cover slips were coated in a 1% Alcian blue 8 GX dye solution at low heat. Following sorting, cells were affixed to cover slips and incubated in a humid chamber. Cells were stained for visualization by immunofluorescence confocal microscopy as follows. Fixed cells were incubated directly with fluorescently conjugated Gr-1, CD11b or F4/80 antibodies followed by the permeabilization using BD Cytofix/Cytoperm buffer (BD Pharmingen). For iNOS staining, FITC-conjugated iNOS (BD Pharmingen) was used in (1:100) in BD block/perm buffer. Stained slides were mounted with the ProLong® Gold antifade reagent with DAPI (Molecular Probes) and analyzed by Zeiss LSM-510 laser scanning confocal microscope. Tissue sections of formalin-fixed and paraffin-embedded 4T-1 tumors were deparaffinized and re-hydrated. Antigens were retrieved using microwave irradiation in citrate buffer pH 6.0 for 15 minutes. Endogenous peroxidase activity was inhibited with 3% (vol/vol) H2O2 in methanol, and nonspecific binding of antibodies was blocked with 1% (wt/vol) BSA for 30 min at room temperature. Tissue sections were incubated overnight with rabbit anti-iNOS (1:1000; Thermo Scientific). Specimens were incubated with horseradish peroxidase (HRP)-conjugated anti-rabbit IgG (Dako) for 30 minutes at room temperature. Peroxidase activity was developed using 3,3-diaminobenzidine tetrahydrochloride (Dako) and H2O2. Hematoxylin was used as a nuclear counter stain in tissue sections. Stained slides were dehydrated and mounted with Cytoseal* 60 (Richard-Allan scientific).

### *In vitro* cell culture experiments

iNOS^+^ cells were isolated as described above. They were resuspended in MLR media (DMEM plus 5% FBS with 10 mM HEPES [pH 7.4], 1% sodium pyruvate, 1% penicillin/streptomycin, 1% l-glutamine, 0.4% L-arginine HCl, 1% folic acid/l-asparagine, and 0.2% 2-ME). *In vitro* suppression assay was carried out as previously described [16]. For detection of apoptosis, cells were first stained for the CD8 antigen and then with anti-Annexin V- allophycocyanin (APC) Ab according to the manufacturer’s protocol (Annexin V apoptosis detection kit; BD Pharmingen) [21]. For nitrite quantification iNOS^+^ cells were cultured (1 × 10^6^ cells/ml) in the presence of recombinant mouse IFN-γ (20 ng/ml) for 6–12 h. A Griess reagent system kit (Promega) was used according to the manufacturer's instructions. Briefly, 50 μl of culture supernatant were added to the plate, followed by the addition of 50 μl of sulfanilamide solution (10 min) and 50 μl of naphthylethylenediamine dihydrochloride (NED) (10 min). Absorbance at 540 nm was measured using a Biotekmicroplate reader and compared to a standard nitrite curve ranging from 0–100 μM.

### Statistical analysis

Student's *t* test was used for comparison between groups in all of the experiments. In all analyses, *P* ≤ 0.05 was considered significant.

## Results

### Identification of an iNOS^+^ subset among the tumor-resident myeloid cell populations

**Figure 1:**
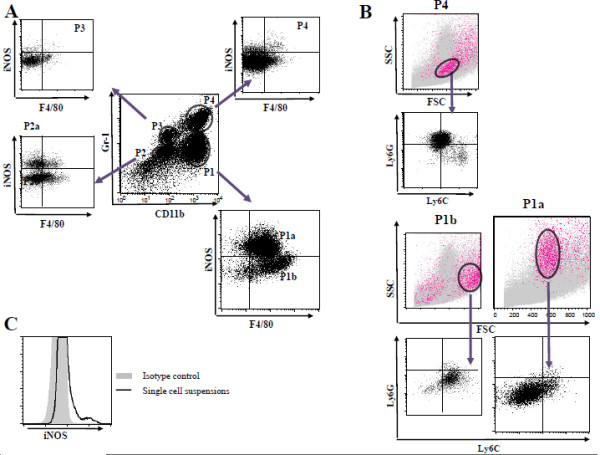
** Identification of iNOS**^**+**^**cells in 4 T-1 tumor.A**. Single-cell suspensions were prepared from established tumors. FACS analysis of tumor-resident heterogeneous myeloid cell populations identified four distinct subsets by the expression of the surface markers CD11b and Gr-1. Expression of iNOS coupled with F4/80 was evaluated within each gated population. The P1 subset was divided into two subfractions P1a and P1b based on iNOS expression **B**. Ly6G/C and FSC/SSC dot plots are shown for gated P1a, P1b and P4 cell subsets. A back gating strategy revealed distinct locations for these subsets on a dot plot graph based on their FSC and SSC profile. P1a (F4/80^+^iNOS^+^), P1b (F4/80^+^iNOS^-^), and P4 (F4/80^-^CD11b^hi^) subsets were further evaluated based on Ly6G andLy6C expression. **C**. The isotype antibody staining (gray filled area) from single-cell suspensions for iNOS is shown.

In the tumor microenvironment, NO activity by infiltrating myeloid cells has been suggested to represent a mechanism for their immunosuppressive properties. Of the three isoforms of NOS which produce NO, inducible (iNOS), endothelial (eNOS) and neuronal (nNOS), only iNOS produces high amounts of NO [24]. In the great majority of the previous studies, NO production by iNOS was monitored via qRT-PCR, immunohistochemistry, Western blotting analysis, or *in vitro* NO production. A few studies examined intracellular iNOS production by flow cytometry in tumor-infiltrating or peripheral cells [9,25], but did not further trace it back to the origin and see whether an iNOS based categorization strategy would lead to a distinct myeloid cell subset. To determine whether iNOS^**+**^ cells constitute a distinct subset among heterogeneous tumor-resident myeloid cell populations, single-cell suspensions from primary tumors were stained for extracellular markers CD11b, Gr-1 and F4/80 followed by intracellular iNOS staining. Figure 1 depicts representative flow cytometry panels identifying intratumoral iNOS^**+**^ cell subsets. Among four different tumor-infiltrating DP myeloid subpopulations, only two subsets; P1a; CD11b^hi^ Gr-1^dim^ F4/80^+^ cells and P2a; CD11b^lo^ Gr-1^dim^ F4/80^-^ cells stained positive for iNOS whereas the Gr-1^int^ CD11b^int^ F4/80^-^ (P3) and the Gr-1^hi^ CD11b^hi^ F4/80^-^ (P4) populations did not (Figure 1A). Since more than 95% of the total iNOS was made by P1a subset, we focused only on this particular subpopulation and the other myeloid subsets, i.e. P1b, P3 and P4 were not pursued further in this study as they did not express iNOS. A back gating analysis of DP subsets based on their forward and side scatter (FSC/SSC) profile revealed P1a, P1b and P4 cells as distinct populations on a dot plot graph (Figure 1B). Although P1a and P4 subsets showed comparable size as measured by FSC, their granularity level based on SSC differed considerably. Because the Gr-1 Ab recognizes both Ly6C and Ly6G epitopes, iNOS and F4/80 gated P1 and CD11b and F4/80 gated P4 subpopulations were further characterized separately with anti-Ly6C and anti-Ly6G Abs. We found P1a, P1b, P4 subsets displayed Ly6G^-^ Ly6C^dim^, Ly6G^-^ Ly6C^hi^, Ly6G^hi^ Ly6C^int^ phenotypes, respectively (Figure 1B). The P4 subset carrying CD11b^hi^ Gr-1^hi^ Ly6G^hi^ and ly6C^int^ corresponded to the classical PMN phenotype [reviewed in Ref.[1-3]. The P1b subset, equivalent to the P1b population in Figure 1A, on the other hand, was positive for MHCII and CXCR4 (data not shown). As previously described by Movahedi et al., these cells with the SSC^low^F4/80^+^Ly6C^hi^CCR3^-^ phenotype were defined as tumor-induced monocytes which can be progenitors of TAM *in vivo*[5]. In contrast, CD11b^hi^ Gr-1^dim^ and F4/80^+^P1a cells expressed Ly6C weakly and did not match the previously described MO-MDSC [1-3]. Histogram with isotype control for iNOS is shown in Figure 1C. We also detected iNOS^+^ P1a subset in the tumors of three other distinct models; implantable CT26 colon carcinoma, B16 melanoma and transgenic spontaneously arising FVBneuN (Additional file 1: Figure S1A). 

### Accumulation kinetics and quantification of iNOS^+^ P1a subset

**Figure 2:**
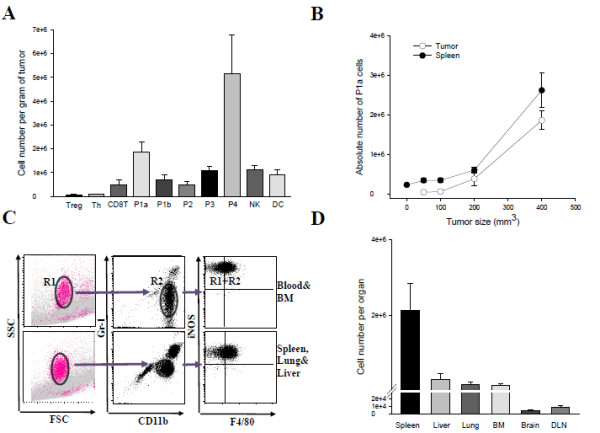
** Accumulation kinetics and quantification of iNOS**^**+**^**subset (P1a).A**. Primary tumors were 400 mm^3^ in size when they were harvested and weighed. The absolute numbers of tumor-infiltrating leukocytes were determined by flow cytometry. Results are expressed as the average number of cells per gram of tumor tissue and were obtained from two separate experiments with 4–5 mice. Error bars represent mean and SD **B**. To determine the time course of expansion of the iNOS^+^ cell subset in the tumor and spleen, mice with various tumor sizes (from 50 mm^3^ to 400 mm^3^) were sacrificed and the absolute number were calculated per gram of tumor tissue or per spleen. **C**. In all of the tested samples the iNOS^+^ cell subset (gated on R1 + R2) was identified as CD11b^+^ Gr-1^dim^ cells (R2) within SSC^hi^ subfraction (R1). **D**. The quantification of the iNOS^+^ cell subset was carried out following the gating strategy shown in C. Error bars = SD, *n* = 4–5 mice per group. The above experiments were repeated twice with similar results.

Next, we wanted to determine the prevalence of the iNOS^**+**^ subset in the tumor and the periphery and whether their accumulation was dependent on tumor growth. As can be seen in Figure 2A, P1a and P4 populations represented the great majority of the tumor-infiltrating leukocyte in comparison to regulatory T- (Treg; CD4^+^ Foxp3^+^), Thelper (Th; CD4^+^ Foxp3^-^), CD8^+^ T, Dendritic (DC; CD11c^+^ MHC II^+^) and Natural Killer (NK; CD3^-^ NKG2D^+^ DX5^+^) cells. Specifically, the average absolute number of P1a subset (~1.85 ×10^6^/g of tumor) was >2-fold higher than the P1b, DC, NK and P3 subsets, >3.5-fold higher than P2 and CD8^+^ T-cells and 20-fold higher than Treg cells. Analysis of the iNOS^**+**^ P1a subset infiltration kinetics revealed that accumulation of these cells was completely dependent on tumor growth (Figure 2B). Their expansion was gradual during early tumor growth (up to 200 mm^3^) but increased rapidly thereafter. The same trend was also observed in the spleen. Significant accumulation of CD11b^hi^Gr-1^dim^ F4/80^+^ and iNOS^**+**^ cells was observed after tumor induction, increasing from 1% of all splenocytes (2.4 × 10^5^ ± 45 × 10^3^) to 4-6% of cells (2.6 ×10^6^ ± 8.5 ×10^5^) at a tumor size of 400 mm^3^. In separate experiments, we further evaluated the presence of iNOS^+^ subset in different peripheral organs such as liver, lung, brain and tumor draining lymph nodes (TDNLs) along with bone marrow and blood of tumor-bearing mice by gating only on high side-scatter subset in single-cell suspensions. As seen in Figure 2C, cells exhibiting SSC^hi^ were clearly distinguishable and among the R1 gated cells, the CD11b^+^ Gr-1^dim^ subset was composed entirely of the iNOS^+^ subset (gated on R1 + R2); validating the back gating strategy shown in the prior figure. The highest relative number of iNOS^+^ cells were detected in the spleen (2.1 × 10^6^ ± 4.5 × 10^4^) compared to lowest number in the DNLs (7 × 10^3^ ± 2.1 × 10^3^) and the brain (4 × 10^3^ ± 7 × 10^2^) (Figure 2D). They were also found in the liver (3 × 10^5^ ± 1.3 × 10^4^), lung (1.5 × 10^5^ ± 5.5 × 10^4^), BM (1.3 × 10^5^ ± 3.4 × 10^4^ per femur & tibia) and blood (1.4 × 10^4^ ± 8.4 × 10^3^/200 cc). Their presence in the BM and blood suggested that they originated in the BM and circulated to the major sites through blood.

### Phenotypic characterization of tumor-infiltrating iNOS^+^ P1a subset

In the past several years numerous studies attempted to categorize immune suppressive MDSC subpopulations based on various combinations of cell surface markers.Murine MO-MDSC have been classified as CD11b^+ ^Ly6G^-^ Ly6C^hi^ cells that express lower levels of F4/80 and higher levels of Gr-1 compared to TAM [1]. Both MDSC and TAM have been found to be positive for IL‐4 receptor‐α (CD124) and M‐CSF receptor (CD115) [1]. Other studies suggested that MO-MDSC represented a mixture of myeloid cells in varying stages of differentiation, from less differentiated to terminally differentiated [9,11,26,27]. In order to link iNOS expression to a particular phenotype, we determined the overall differentiation/maturation stage and further characterized iNOS^+^ P1a cells based on the differential expression of selected phenotypic markers. To be able to compare the relative expression level of each marker, we also included the P4 subset as a control. iNOS^+^ cells did not express typical neutrophils markers CXCR2 (chemokine receptor for neutrophils) or Ly6G in contrast to the iNOS^**-**^ P4 subset (Table 1). Therefore, these cells were distinct from PMN as described in literature [1-3]. They were also negative for CCR3 which is a typical chemokine receptor for eosinophils. iNOS^+^ cells however, expressed low levels of Siglec-F which is found on immature cells of the myelomonocytic lineage and eosinophils. Moreover DC markers such as CD11c, MHC II and CD86 were absent (Additional file 1: Figure S1C). Ly6C, a marker that has been reported to be associated particularly with MO-MDSC, was weakly-expressed on the iNOS^+^ cell subset. It has been hypothesized that monocytic-like MDSC (CD11b^+^ Gr-1^lo^ and F4/80^lo^ CD124^+^) could differentiate into F4/80^+^ TAM in tumor microenvironment [5,28]. To determine whether the iNOS^+^ cell subset belongs to TAM or MO-MDSC we included two other markers, CD115& CD124 that are co-expressed by those cells [1]. P1a subset was found to be negative for both of these markers (Table 1&Additional file 1: Figure S1C). Thus, these data suggested that iNOS^+^ P1a subset displayed a phenotype that is not consistent with that of MO-MDSC or TAM and therefore are unlikely to be of monocytic origin. P4 subset however, differentially expressed CD309 (VEGFR2), CD86 (B7-2), CD138 (Syndecan-1) and CD124. Together, these results show that the iNOS^+^ cells were phenotypically distinct from PMN as well as MO subsets and shared few markers with TAM but could not be classified into any standard MDSC subset. 

**Table 1:** **Phenotypic analysis of tumor infiltrating iNOS**^**+**^**P1a and negative P4 subpopulations**

	**P4**	**P1a**
Monocyte/PMN subset marker/Migration		
Ly6C	+	-/+
Ly6G	++	-
CXCR2	++	-
CCR3	+	-
Siglec-F	-	-/+
F4/80	-	++
CXCR4	-	-
CD62L	-	-
CD49d	++	++
Adhesion/Activation Molecules		++
CD44	++	++
CD43	++	-
CD103	-	-
CD138	++	++
CD54 (ICAM-1)	++	++
Antigen presentation		-
MHC I	++	-
MHC II	-	-
CD86 (B7-2)	+	+
CD11c	-	-
Differentiation		-
CD119 (IFNGR1)	++	-
CD124 (IL-4Ra)	++	+
CD115 (CSFR1)	-	-
CD309 (VEGFR2)	+	+
Potential T-cell suppressive marker		
B7H1 (PD-L1)	+	+
B7DC (PD-L2)	-	-
FASL	+	-
Pattern recognition receptor		-
CD14	-	-
TLR2	+	+ +

Expression of the indicated cell surface markers was evaluated on gated SSC^hi^ CD11b^hi^ Gr-1^dim^ F4/80^+^ P1a and Gr-1^hi^ CD11b^hi^ F4/80^-^ (P4) cells. “-” indicates no expression and “+”, “++”, increasing amount of expression to isotype-matched controls.

### Morphology, intracellular structure and localization of iNOS^+^ ring cells in the tumor

**Figure 3:**
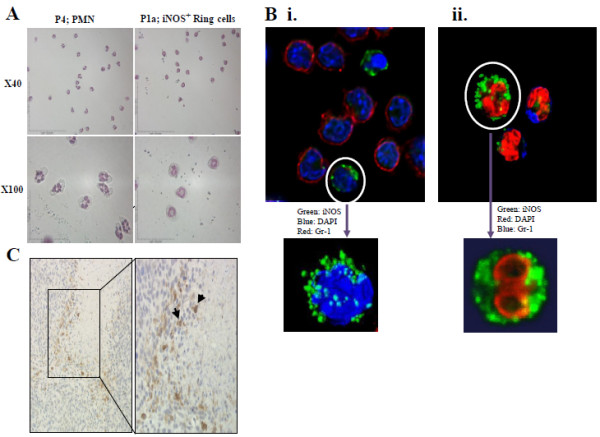
** Morphology and immunohistochemistry analysis of iNOS**^**+**^**cells*.*A**. Tumor-purified iNOS^+^ (P1a) and iNOS^-^ (P4 = PMN) fractions were first evaluated by cytospin followed by Wright-Giemsa staining. Pictures are shown in two different magnifications (x40 and x100). Data are representative of at least two experiments. **B**.**Bi**: Confocal microscopy optical section of a mixture of PMN and iNOS^+^ ring cells, with red representing Gr-1, green iNOS and blue nuclear (DAPI) staining. Inset: 3D volume rendering of an iNOS^+^ cell demonstrating iNOS-positive vesicles surrounding the nucleus. **Bii**: 3D volume rendering derived from a stack of confocal optical sections with green representing iNOS staining, red as DAPI and blue as Gr-1 staining. Inset: 3D volume rendering of a single iNOS^+^ cell showing the torus shaped nucleus as seen after using clipping planes to "remove" half of the volume along the z-axis. The nucleus in this cell is orientated at right angles to those seen in A (x100). **C**. Histological analysis of a section from a primary tumor. The necrotic areas could be differentiated by debris. The results are representative of two independent experiments.

To confirm that P1a did not represent a mononuclear cell subset iNOS^+^ P1a and iNOS^-^ P4 subsets were evaluated for their morphology using cytospin analysis. The subsets were first enriched from tumors by magnetic bead technology followed by cell sorting using the protocol described in material and methods. Wright-Giemsa staining of sorted preparations demonstrated a polymorphonuclear morphology for P4 subset cells (Figure 3A). In contrast, most of the cells of the P1a have displayed ring-shaped nuclei, a unique morphology distinct from monocytes and macrophages. This type of morphology with a comparable phenotype has been reported in *in vitro* generated MDSC as well as in different murine models of inflammation, traumatic stress, parasitic infections, and cancer [29-33] Although cytospin analysis distinguished this subset from PMN based on nuclear staining, it did not show the high level of granularity that would be predicted by the high side-scatter pattern observed in flow analysis (Figure 1A). Confocal microscopy analysis of immuno stained cells isolated from tumors revealed intense punctate iNOS-staining within the cytoplasm adjacent to the cell membrane, consistent with the presence of iNOS in pre-formed vesicles (Figure 3Bi-ii&insets). Rapid tumor cell proliferation causes hypoxic/necrotic areas and F4/80^+^ cells have been especially shown to accumulate rapidly in hypoxic regions of tumors [reviewed in Refs. [34,35]. To this end, we wanted to examine the distribution of iNOS^+^ ring cells within the tumor in a series of immunohistochemistry sections of tumor tissues. Histological analysis of a section from a primary tumor illustrated a central area of necrosis with iNOS^+^ ring cells being localized predominantly at the periphery of this area (Figure 3C). 

### Functional analysis of iNOS^+^ ring subset

**Figure 4:**
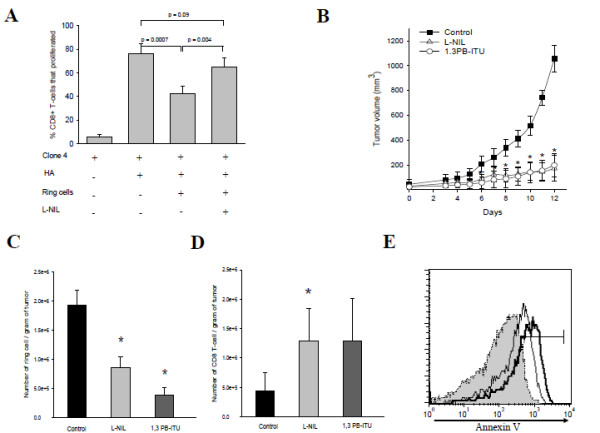
** Functional analysis of iNOS**^**+**^**ring subset.A**. CFSE-labeled Clone 4 transgenic CD8^+^ T-cells stimulated with HA-peptide pulsed DC co-cultured in the presence or absence of sorted ring cells from 4T1 tumors. Selective iNOS inhibitor L-NIL was added to some wells. T cell proliferation was measured by CFSE dilution assay using flow cytometry. Data are plotted as percent CD8^+^ T-cells proliferation and are from one of three independent experiments. Error bars show SD. **B**. Effect of selective iNOS inhibitors L-NIL and 1,3-PB-ITU on tumor growth. Mice were treated with L-NIL or 1,3-PB-ITU via daily i.p injections starting one day after 4T-1 injection. Tumor volume was determined daily using the formula a^2^ x b/2, where a and b are the shortest and longest perpendicular dimensions of the tumor, respectively. * The differences between the L-NIL or 1,3-PB-ITU-treated group and control (4T-1 only) were significant (p <0.0004 on days 6–15). Error bars = SD, n = 5–7 mice per group. Representative of 2 independent experiments. **C**&**D**. Intratumoral Ring and CD8^+^ T-cells numbers were quantified by flow cytometric analysis of day 9 tumors. Graphs represent the mean (±SD) of at least 5 mice from 2 independent experiments. D *The differences between the L-NIL- treated and control groups was significant (P ≤ 0.05), the differences between the 1,3-PB-ITU-treated group and control was not (p <0.1). **E**. The ability of Ring cells to kill CD8^+^ T-cells was evaluated in an *in vitro* co-culture assay. Ring cells were sorted and added at different ratios with CD8^+^ T-cells and apoptosis of CD8^+^ T-cells was measured via staining with Annexin V. The apoptosis effect was related to ring cell abundance. The gray histogram represents CD8^+^ T-cells alone versus thin open histogram (1:1 ratio of CD8^+^ T/Ring) and thick open histogram (1:2 ratio)Representative of 3 independent experiments.

T cell-suppressive activity through iNOS- or ARG-mediated mechanisms is a prominent feature of MDSC [1-3]. To verify whether iNOS^+^ ring cells accumulating in the tumor were immunosuppressive, ring cells were sorted from tumors and tested for *in vitro* NO production and T-cell proliferation assay. The live gate (CD45^+^ 7-AAD^-^) from single-cell suspensions and the expression of iNOS in pre- and post-sort cells are shown in Additional file 1: Figure S1B. iNOS expression overlapped with the appearance of significant nitrite concentrations in the cultures, indicative of high NO production (Additional file 1: Figure S1D). iNOS^+^ ring cells were co-cultured with clone4 CD8^+^ T-cells expressing a TCR specific for the influenza virus hemagglutinin (HA) at a 1:1 ratio for 48 hours in the presence of HA peptide. As shown in Figure 4A, they inhibited the proliferation of CD8^+^ T-cells by 2-fold in an iNOS-dependent manner, as addition of the iNOS inhibitor L-NIL into culture restored T-cell proliferation. Similarly, we tested selective iNOS inhibitors L-NIL and 1,3-PB-ITU *in vivo* to determine whether NO was critical to tumor progression. Mice were treated with L-NIL or 1,3-PB-ITU (both potent and selective inhibitor of iNOS) via daily i.p injections starting from day 1 following 4T-1 injection (Figure 4B). *In vivo* blocking of iNOS activity with these inhibitors significantly inhibited the overall rate of tumor growth when compared with the 4T-1 cells alone group (control). Furthermore, L-NIL showed the same effect in another model; B16 melanoma. Every day treatment starting from the day of tumor cell inoculation and up to 24 days attenuated tumor growth (Additional file 1: Figure S1E). Superior tumor regression in L-NIL or 1,3-PB-ITU-treated mice was associated with a decreased number of ring cells both in tumor (Figure 4C) and spleen (data not shown) on day 9 after tumor injection. The highly significant decrease in iNOS^+^ ring cell accumulation kinetic was accompanied by an enhancement of tumor-resident CD8^+^ T-cell quantity in both of the iNOS inhibitor-treated groups (Figure 4D). Finally, the effect of tumor-derived iNOS^+^ ring cell on CD8^+^ T-cell survival was evaluated in an *in vitro* co-culture assay. Ring cells purified from tumor were cultured with CD8^+^ T-cell sorted magnetically from the DNLs of the same mice for 24–48 h. The histogram data shown in Figure 4E demonstrate that Ring cells induced CD8^+^ T-cell apoptosis depending on the cell number as detected by Annexin V staining. 80 ± 10% of CD8^+^ T-cells became apoptotic in the co-cultures at 1:2 ratio (CD8^+^ T/Ring cell) compared to 60 ± 15% at 1:1 ratio. 

## Discussion

Immature myeloid cells are a heterogeneous population and include precursors of granulocytes, macrophages and DC. MDSC, a sub-population of immature myeloid cells, have been defined primarily by their immune suppressive activity. In mice, MDSC have been characterized by the co-expression of CD11b and Gr-1 antigens. They can weakly express mature myeloid cell markers such as CD11c, F4/80 and MHC class II. However, none of these cell surface markers is definitive since their expression varies based on inducing tumor. Absence of a unique marker that is specific to MDSC has often led to diverse and sometimes contradictory descriptions of the subpopulations. On the other hand it has been well established that MDSC metabolize L-arginine as a result of increased ARG or iNOS expression making these proteins hallmarks of suppression. The great majority of the early *in vitro* and *in vivo* studies on the origins and function of these cells utilized indirect methods for iNOS detection. In this study we asked whether iNOS could serve as a specific marker in the direct identification of a homogenously distributed subset of IMC. Among tumor-infiltrating DP myeloid populations iNOS expression was primarily detected in the CD11b^+^ F4/80^+^ Gr-1^dim^subset; a common phenotype shared mostly by TAM [1,9] and to some extent MO-MDSC, which are the potential progenitors of strongly suppressive macrophages. A similar phenotype, SSC^hi^ F4/80^+^ Gr-1^int^CD11c^-^ was also used to describe eosinophils [7,9], PMN [10,33] and inflammatory monocytes [36,37] in different studies. Because of the close phenotypic resemblance, we screened iNOS^+^ cells with a large set of different markers known to be associated with different cell populations. The F4/80^+^ iNOS^+^ P1a cells were phenotypically different from macrophages/monocytes and MO-MDSC in that they weakly expressed Gr-1 and Ly6C, and most importantly were negative for CD115, CD124 and CXCR4 [1,26,38]. P1a cells were not eosinophils since they did not express CCR3 and weakly expressed Siglec-F which is a marker for immature cells of the myelomonocytic lineage. They also lacked neutrophil markers Ly6G and CXCR2 [1,26].We also tested the expression of CD49d which was suggested as an alternative marker for Gr-1 when used together with CD11b [10]. In our tumor model, CD49d did not distinguish between the PMN and iNOS^+^ cells as both the P4 and P1a subsets expressed it equivalently (Table 1). Overall, iNOS^+^ P1a cells could not be phenotypically classified into any MDSC subset described here. However, morphological analysis by Wright-Giemsa staining revealed the presence of ring-shaped nucleus suggesting a PMN-like etiology (Figure 3A). Ring-shaped nuclei were previously described as a characteristic of immature neutrophils as opposed to the typical segmented multilobular nucleus detected in polymorphonuclear neutrophils [33,39]. Granulocytes and macrophages differentiate from a common, committed progenitor cell. It has been previously shown that signals that lead to myelopoiesis affect the maturation process and cause the accumulation of cells which retain their neutrophil-like ring nuclei while acquiring macrophage differentiation markers such as F4/80 on the cell surface in the BM [30,40].

To achieve a detailed description of the ring cells, tumor-isolated cells were further analyzed in 3D by confocal microscopy. F4/80^+^ iNOS^+^ P1a cells represent an immature stage of neutrophil maturation with incompletely condensed, non-segmented torus-shaped nuclei (dumbbell-shaped cross section) (Figure 3Bii, inset). We detected BM-derived ring cells in diverse peripheral locations but found them excessively in the tumor and spleen. Analysis of their kinetics in both tumor and spleen showed that accumulation of these cells was completely dependent on tumor growth indicating the role of tumor-derived factors in their generation and prevalence. Their presence in the circulation suggests that they migrate via blood however we cannot rule out the possibility that they may also expand directly in organs due to extra medullary hematopoiesis which has been observed in both inflammatory diseases and cancer [32,41].

The presence of ring cells in the BM as myeloid precursor cells with ring-shaped nuclei and to some extent in the peripheral organs has been reported; however this population had not been isolated to uniformity or characterized and assigned a particular function. Premature tumor-infiltrating ring cells may play an essential role in establishment of tumor immuno supression by decreasing T-cell proliferation and/or survival via NO production. Our findings are consistent with this notion such that inhibition of iNOS by selective inhibitors resulted in an increase in intratumoral CD8 T-cell numbers and enhanced tumor suppression. Importantly, the inhibition of iNOS also resulted in a significant reduction of intratumoral ring cells without inducing their apoptosis (data not shown). This finding that suggests that in addition to its immune suppressive activity NO is required for the accumulation of ring cells in tumors. When we examined the distribution of iNOS^+^ ring cells within the tumor by histology, the areas of strong iNOS expression were observed to be associated with highly necrotic areas (Figure 3C). These data were consistent with earlier studies that had shown iNOS^+^ cells infiltrated into and around the necrotic areas in different disease models [42,43]. This pattern of localization occurs presumably because of hypoxia-inducible factors which stimulates iNOS^+^ cell accumulation around hypoxic/necrotic areas, which is also linked to other events such as angiogenesis and metastasis [29,44].

In our previous study we had investigated the specific role of NO in IL-12-mediated tumor regression in a lung carcinoma model and we demonstrated that NO was a significant impediment to IL-12 immunotherapy in mice with established tumors [19]. In that study, the source of NO was traced to the TAM-like, CD11b^+^ Gr-1^lo^ F4/80^+^ iNOS^+^subset which had been associated with post-IL-12 NO production however these cells were not characterized further. The current study identifies this subset as a unique MDSC population (ring cells) distinct from TAM or MO-MDSC and more akin to PMN-MDSC. On the other hand, these data cannot rule out the possibility that iNOS^+^ subset include the precursors of inflammatory M1 type classical macrophages or DCs. *In vitro* manipulation studies to differentiate these cells into mature myeloid cells are currently ongoing.

## Conclusion

Together our data provide novel insights for iNOS expressing MDSC and suggest iNOS as a marker to identify a particular subset. The greatest barrier to fully describing IMC between disease models and between species lies in the lack of appropriate phenotypic markers, and mechanistic studies. Classification of MDSC subsets based on unique functional markers may simplify their analysis and lead to the design of functionally-targeted superior immunotherapeutic strategies. In this study, iNOS expression was linked to homogenously distributed ring cells with a particular phenotype and immune suppressive function. To our knowledge, this is the first report to reveal the functional identity of tumor-infiltrating ring cells. Since the absence of a Gr-1 homolog in humans has made the identification of MDSC much more challenging, the use of iNOS as a functional marker of MDSC may also have clinical importance.

## Abbreviations

MDSC: Myeloid-derived Suppressor Cells; IMC: Immature myeloid cells; iNOS: inducible nitric oxide synthase; TAM: Tumor-associated macrophages; TIL: Tumor-infiltrating leukocyte; DC: Dendritic cells; ARG: Arginase; PMN: Polymorphonuclear; MO: Mononuclear; BM: Bone marrow; NO: Nitric oxide; TDNLs: Tumor draining lymph nodes.

## Competing interests

The authors declare that they have no competing interests.

## Authors' contributions

LPV, JLH and PS carried out the experiments. WJS and FY contributed to method development. NKE and BM assisted with data interpretation, supplies and edited the manuscript. MOK designed, supervised the study and wrote the manuscript. All authors read and approved the final manuscript.

## Supplementary Material

Additional file 1 ** Figure 1.** Panel A. Tumor-infiltrating F4/80+ iNOS+ cells in single-cell suspensions from three other distinct tumor models are shown. Panel B. Tumor infiltrated single viable cells were identified as CD45+ and 7-AAD-. Next, histogram analysis for iNOS expression on pre-sort live cells (dashed line) and post-sort cells (bold line) is shown. All cells are SSChi and F4/80+ . Gray filled in peaks represent isotype control. Panel C. Sorted iNOS+ cells (black line) were analyzed for the expression of mature APC markers (CD11c, MHCII, CXCR4, CD124) and Siglec-F relative to isotype controls (gray filled). Panel D. iNOS+ cells were isolated from the spleen and plated. Supernatants were collected after 6-12 h and analyzed for nitrite concentration. Columns, mean of triplicate wells with SD. Experiment was repeated two times with equivalent results. Panel E. Effect of selective iNOS inhibitor L-NIL on B16 tumor growth. Every day treatment starting from the day of tumor cell inoculation and up to 24 days attenuates B16 melanoma tumor growth. Click here for file

## References

[B1] Gabrilovich DI Nagaraj S Myeloid-derived suppressor cells as regulators of the immune system Nat Rev Immunol 2009 9 162 174 10.1038/nri2506 19197294 PMC2828349

[B2] Dolcetti L Marigo I Mantelli B Peranzoni E Zanovello P Bronte V Myeloid-derived suppressor cell role in tumor-related inflammation Cancer Lett 2008 267 2 216 212 10.1016/j.canlet.2008.03.012 18433992

[B3] Talmadge JE Pathways mediating the expansion and immunosuppressive activity of myeloid-derived suppressor cells and their relevance to cancer therapy Clin Cancer Res 2007 13 5243 5248 10.1158/1078-0432.CCR-07-0182 17875751

[B4] Gabrilovich DI Bronte V Chen SH Colombo MP Ochoa A Ostrand-Rosenberg S Schreiber H The terminology issue for myeloid-derived suppressor cells Cancer Res 2007 67 (1 425 17210725 10.1158/0008-5472.CAN-06-3037 PMC1941787

[B5] Movahedi K Guilliams M Van den Bossche J Van den Bergh R Gysemans C Beschin A De Baetselier P Van Ginderachter JA Identification of discrete tumor-induced myeloid-derived suppressor cell subpopulations with distinct T cell-suppressive activity Blood 2008 111 4233 4244 10.1182/blood-2007-07-099226 18272812

[B6] Youn JI Nagaraj S Collazo M Gabrilovich DI Subsets of myeloid-derived suppressor cells in tumor-bearing mice J Immunol 2008 181 5791 5802 18832739 10.4049/jimmunol.181.8.5791 PMC2575748

[B7] Greifenberg V Ribechini E Rössner S Lutz MB Myeloid-derived suppressor cell activation by combined LPS and IFN-gamma treatment impairs DC development Eur J Immunol 2009 39 10 2865 2876 10.1002/eji.200939486 19637228

[B8] Dolcetti L Peranzoni E Ugel S Marigo I Fernandez-Gomez A Mesa C Geilich M Winkels G Traggiai E Casati A Grassi F Bronte V Hierarchy of immunosuppressive strength among myeloid-derived suppressor cell subsets is determined by GM-CSF Eur J Immunol 2010 40 22 35 19941314 10.1002/eji.200939903

[B9] Movahedi K Laoui D Gysemans C Baeten M Stangé G Van den Bossche J Mack M Pipeleers D In't Veld P De Baetselier P Van Ginderachter JA Different tumor microenvironments contain functionally distinct subsets of macrophages derived from Ly6C(high) monocytes Cancer Res 2010 70 14 5728 5739 10.1158/0008-5472.CAN-09-4672 20570887

[B10] Haile LA Gamrekelashvili J Manns MP Korangy F Greten TF CD49d is a new marker for distinct myeloid-derived suppressor cell subpopulations in mice J Immunol 2010 185 1 203 210 10.4049/jimmunol.0903573 20525890

[B11] Laoui D Van Overmeire E Movahedi K Van-den-Bossche J Schouppe E Mommer C Nikolaou A Morias Y De-Baetselier P VanGinderachter JA Mononuclear phagocyte heterogeneity in cancer: different subsets and activation states reaching out at the tumor site Immunobiology 2011 216 11 1192 1202 10.1016/j.imbio.2011.06.007 21803441

[B12] Youn JI Gabrilovich DI The biology of myeloid-derived suppressor cells: the blessing and the curse of morphological and functional heterogeneity Eur J Immunol 2010 40 11 2969 2975 10.1002/eji.201040895 21061430 PMC3277452

[B13] Li Q Pan PY Gu P Xu D Chen SH Role of immature myeloid Gr-1+ cells in the development of antitumor immunity Cancer Res 2004 64 3 1130 1139 10.1158/0008-5472.CAN-03-1715 14871848

[B14] Yamamoto Y Ishigaki H Ishida H Itoh Y Noda Y Ogasawara K Analysis of splenic Gr-1int immature myeloid cells in tumor-bearing mice Microbiol Immunol 2008 52 1 47 53 18352913 10.1111/j.1348-0421.2008.00009.x

[B15] Montero AJ Diaz-Montero CM Kyriakopoulos CE Bronte V Mandruzzato S Myeloid-derived suppressor cells in cancer patients: a clinical perspective J Immunother 2012 35 2 107 115 10.1097/CJI.0b013e318242169f 22306898

[B16] Jamie LH Tao G Mehmet OK Lauren PV Rowswell-Turner RB Nejat KE Dichotomous Effects of IFN-Gamma on Dendritic Cell Function Determine the Extent of IL-12-driven Antitumor T-cell Immunity J Immunol 2011 187 1 126 132 10.4049/jimmunol.1100168 21632715 PMC3119751

[B17] Gu T Rowswell-Turner RB Kilinc MO Egilmez NK Central Role of IFNγ-Indoleamine 2,3 Dioxygenase Axis in Regulation of Interleukin-12- mediated antitumor immunity Cancer Res 2010 70 1 129 138 10.1158/0008-5472.CAN-09-3170 20028855 PMC2805056

[B18] Kilinc MO Aulakh KS Nair RE Jones SA Alard P Kosiewicz MM Egilmez NK Reversing Tumor Immune Suppression with Intra-tumoral IL-12: Activation of Tumor-Associated T-Effector/Memory Cells, Induction of T-Suppressor Apoptosis and Infiltration of CD8+ T-Effectors J Immunol 2006 177 6962 6973 17082611 10.4049/jimmunol.177.10.6962

[B19] Kilinc MO Mukundan L Esma S Yolcu ES Singh NP Suttles J Shirwan H Generation of a multimeric form of CD40L with potent immunostimulatory activity using streptavidin as a chaperon ExpMolPathol 2006 80 252 261 10.1016/j.yexmp.2005.12.004 16487512

[B20] Egilmez NK Harden JL Virtuoso LP Schwendener RA Kilinc MO Nitric Oxide Short-circuits Interleukin-12-mediated Tumor Regression Cancer Immunol Immunother 2011 60 6 839 845 10.1007/s00262-011-0998-2 21387108 PMC11028488

[B21] Kilinc MO Gu T Harden JL Virtuoso LP Egilmez NK Central role of tumor-associated CD8+ T effect or/memory cells in restoring systemic antitumor immunity J Immunol 2009 182 7 4217 4225 10.4049/jimmunol.0802793 19299720

[B22] Kilinc MO Rowswell-Turner RB Gu T Virtuoso LP Egilmez NK Activated CD8+ T-effect or/memory Cells Eliminate CD4+ CD25+ Foxp3+ T-suppressor cells from Tumors via FasL Mediated Apoptosis J Immunol 2009 183 12 7656 7660 10.4049/jimmunol.0902625 19923444

[B23] Broderick L Bankert RB Membrane-associated TGF-beta1 inhibits human memory T cell signaling in malignant and nonmalignant inflammatory microenvironments J Immunol 2006 177 5 3082 3088 16920945 10.4049/jimmunol.177.5.3082

[B24] Paradise WA Vesper BJ Goel A Waltonen JD Altman KW Haines GK III Radosevich JA Nitric oxide: perspectives and emerging studies of a well-known cytotoxin Int J MolSci 2010 11 2715 2745 10.3390/ijms11072715 PMC2920563 20717533

[B25] Sinha P Okoro C Foell D Freeze HH Ostrand-Rosenberg S Srikrishna G Proinflammatory S100 proteins regulate the accumulation of myeloid-derived suppressor cells J Immunol 2008 181 7 4666 4675 18802069 10.4049/jimmunol.181.7.4666 PMC2810501

[B26] Sawanobori Y Ueha S Kurachi M Shimaoka T Talmadge JE Abe J Shono Y Kitabatake M Kakimi K Mukaida N Matsushima K Chemokine-mediated rapid turnover of myeloid-derived suppressor cells in tumor-bearing mice Blood 2008 111 12 5457 5466 10.1182/blood-2008-01-136895 18375791

[B27] Geissmann F Manz MG Jung S Sieweke MH Merad M Ley K Development of monocytes, macrophages, and dendritic cells Science 2010 327 5966 656 661 10.1126/science.1178331 20133564 PMC2887389

[B28] Sica A Bronte V Altered macrophage differentiation and immune dysfunction in tumor development J Clin Invest 2007 117 5 1155 1166 10.1172/JCI31422 17476345 PMC1857267

[B29] Rössner S Voigtländer C Wiethe C Hänig J Seifarth C Lutz MB Myeloid dendritic cell precursors generated from bone marrow suppress T cell responses via cell contact and nitric oxide production in vitro Eur J Immunol 2005 35 12 3533 3544 10.1002/eji.200526172 16331707

[B30] Ribechini E Leenen PJ Lutz MB Gr-1 antibody induces STAT signaling, macrophage marker expression and abrogation of myeloid-derived suppressor cell activity in BM cells Eur J Immunol 2009 39 12 3538 3551 10.1002/eji.200939530 19830733

[B31] Makarenkova VP Bansal V Matta BM Perez LA Ochoa JB CD11b+/Gr-1+ myeloid suppressor cells cause T cell dysfunction after traumatic stress J Immunol 2006 176 4 2085 2094 16455964 10.4049/jimmunol.176.4.2085

[B32] Van Ginderachter JA Beschin A De Baetselier P Raes G Myeloid-derived suppressor cells in parasitic infections Eur J Immunol 2010 40 11 2976 2985 10.1002/eji.201040911 21061431

[B33] Tsuda Y Takahashi H Kobayashi M Hanafusa T Herndon DN Suzuki F Three different neutrophil subsets exhibited in mice with different susceptibilities to infection by methicillin-resistant Staphylococcus aureus Immunity 2004 21 2 215 226 10.1016/j.immuni.2004.07.006 15308102

[B34] Murdoch C Giannoudis A Lewis CE Mechanisms regulating the recruitment of macrophages into hypoxic areas of tumors and other ischemic tissues Blood 2004 104 8 2224 2234 10.1182/blood-2004-03-1109 15231578

[B35] Lewis CE Pollard JW Distinct role of macrophages in different tumor microenvironments Cancer Res 2006 66 2 605 612 10.1158/0008-5472.CAN-05-4005 16423985

[B36] Biermann H Pietz B Dreier R Schmid KW Sorg C Sunderkotter C Murine leukocytes with ring-shaped nuclei include granulocytes, monocytes, and their precursors J LeukocBiol 1999 65 217 231 10.1002/jlb.65.2.217 10088605

[B37] Dunay IR Damatta RA Fux B Presti R Greco S Colonna M Sibley LD Gr1(+) inflammatory monocytes are required for mucosal resistance to the pathogen Toxoplasma gondii Immunity 2008 29 2 306 317 10.1016/j.immuni.2008.05.019 18691912 PMC2605393

[B38] Yang L Huang J Ren X Gorska AE Chytil A Aakre M Carbone DP Matrisian LM Richmond A Lin PC Moses HL Abrogation of TGF beta signaling in mammary carcinomas recruits Gr-1+CD11b+ myeloid cells that promote metastasis Cancer Cell 2008 13 23 35 10.1016/j.ccr.2007.12.004 18167337 PMC2245859

[B39] Zhu B Bando Y Xiao S Yang K Anderson AC Kuchroo VK Khoury SJ CD11b + Ly-6 C(hi) suppressive monocytes in experimental autoimmune encephalomyelitis J Immunol 2007 179 8 5228 5237 17911608 10.4049/jimmunol.179.8.5228

[B40] Sasmono RT Ehrnsperger A Cronau SL Ravasi T Kandane R Hickey MJ Cook AD Himes SR Hamilton JA Hume DA Mouse neutrophilic granulocytes express mRNA encoding the macrophage colony-stimulating factor receptor (CSF-1R) as well as many other macrophage-specific transcripts and can transdifferentiate into macrophages in vitro in response to CSF-1 J LeukocBiol 2007 82 1 111 123 10.1189/jlb.1206713 17438263

[B41] Kusmartsev SA Li Y Chen SH Gr-1+ myeloid cells derived from tumor-bearing mice inhibit primary T cell activation induced through CD3/CD28 costimulation J Immunol 2000 165 779 785 10878351 10.4049/jimmunol.165.2.779

[B42] Mabuchi A Nagao T Koshio O Ishiwata T Yano A Suzuki K Yokomuro K Wheatley AM Role of F4/80Mac-1 adherent non-parenchymal liver cells in concanavalin A-induced hepatic injury in mice Hepatol Res 2008 38 10 1040 1049 10.1111/j.1872-034X.2008.00362.x 18513334

[B43] Tatemichi M Ogura T Sakurazawa N Nagata H Sugita M Esumi H Roles of inducible nitric oxide synthase in the development and healing of experimentally induced gastric ulcers Int J ExpPathol 2003 84 5 213 220 10.1111/j.1365-2613.2003.00357.x PMC2517565 14690480

[B44] Du R Lu KV Petritsch C Liu P Ganss R Passegué E Song H Vandenberg S Johnson RS Werb Z Bergers G HIF1 induces the recruitment of bone marrow-derived vascular modulatory cells to regulate tumor angiogenesis and invasion Cancer Cell 2008 13 206 220 10.1016/j.ccr.2008.01.034 18328425 PMC2643426

