# Supplementary material for: Characterization of iNOS+ Neutrophil-like ring cell in tumor-bearing mice
Source: J Transl Med. 2012 Jul 30;10:152. doi: 10.1186/1479-5876-10-152 (PMC3478162; doi:10.1186/1479-5876-10-152)
Supplement: Additional file 1 — Figure 1. Panel A. Tumor-infiltrating F4/80+ iNOS+ cells in single-cell suspensions from three other distinct tumor models are shown. Panel B. Tumor infiltrated single viable cells were identified as CD45+ and 7-AAD-. Next, histogram analysis for iNOS expression on pre-sort live cells (dashed line) and post-sort cells (bold line) is shown. All cells are SSChi and F4/80+ . Gray filled in peaks represent isotype control. Panel C. Sorted iNOS+ cells (black line) were analyzed for the expression of mature APC markers (CD11c, MHCII, CXCR4, CD124) and Siglec-F relative to isotype controls (gray filled). Panel D. iNOS+ cells were isolated from the spleen and plated. Supernatants were collected after 6-12 h and analyzed for nitrite concentration. Columns, mean of triplicate wells with SD. Experiment was repeated two times with equivalent results. Panel E. Effect of selective iNOS inhibitor L-NIL on B16 tumor growth. Every day treatment starting from the day of tumor cell inoculation and up to 24 days attenuates B16 melanoma tumor growth. [file 1479-5876-10-152-S1.pptx]

## Slide 1
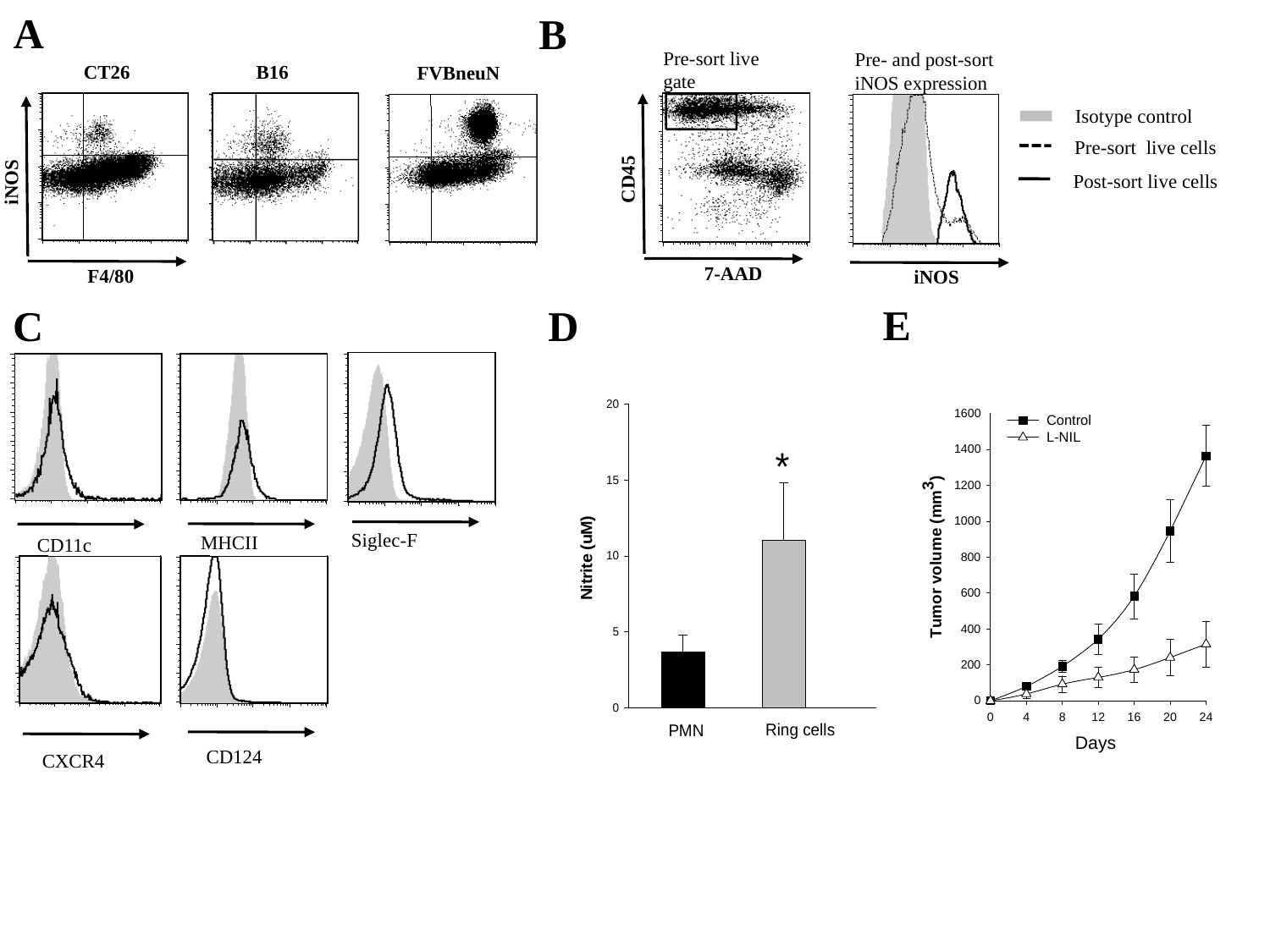

A
B
Pre-sort live gate
Pre- and post-sort iNOS expression
B16
CT26
FVBneuN
CD45
7-AAD
Isotype control
Pre-sort live cells
Post-sort live cells
iNOS
F4/80
iNOS
E
C
D
Siglec-F
MHCII
CD11c
CD124
CXCR4
